# In-Situ Monitoring and Diagnosing for **Fused Filament Fabrication** Process Based on Vibration Sensors

**DOI:** 10.3390/s19112589

**Published:** 2019-06-06

**Authors:** Yongxiang Li, Wei Zhao, Qiushi Li, Tongcai Wang, Gong Wang

**Affiliations:** 1 CAS Key Laboratory of Space Manufacturing Technology, Technology and Engineering Center for Space Utilization, Chinese Academy of Sciences, Beijing 100094, China; liyongxiang@csu.ac.cn (Y.L.); liqiushi17@csu.ac.cn (Q.L.); wangtongcai@csu.ac.cn (T.W.); 2 University of Chinese Academy of Science, Beijing 100049, China

**Keywords:** FFF, in-situ monitoring, machine states, product quality, vibration sensor, data-driven

## Abstract

Fused filament fabrication (FFF) is one of the most widely used additive manufacturing (AM) technologies and it has great potential in fabricating prototypes with complex geometry. For high quality manufacturing, monitoring the products in real time is as important as maintaining the FFF machine in the normal state. This paper introduces an approach that is based on the vibration sensors and data-driven methods for in-situ monitoring and diagnosing the FFF process. The least squares support vector machine (LS-SVM) algorithm has been applied for identifying the normal and filament jam states of the FFF machine, besides fault diagnosis in real time. The identification accuracy for the case studies explored here using LS-SVM is greater than 90%. Furthermore, to ensure the product quality during the FFF process, the back-propagation neural network (BPNN) algorithm has been used to monitor and diagnose the quality defects, as well as the warpage and material stack caused by abnormal leakage for the products in-situ. The diagnosis accuracy for the case studies explored here using BPNN is greater than 95%. Results from the experiments show that the proposed approach can accurately recognize the machine failures and quality defects during the FFF process, thus effectively assuring the product quality.

## 1. Introduction

The fused filament fabrication (FFF) technique is a generic additive manufacturing (AM) technology [1,2,3,4] for thermoplastic materials and has been increasingly used in various fields like aerospace and healthcare [5,6]. The FFF process provides great flexibility in fabricating a three-dimensional (3D) part with complex geometry at a relatively low cost. However, qualifying these parts is challenging due to the open-loop control of the FFF process. There is an urgent need to develop an advanced monitoring and closed-loop quality control methods for monitoring the FFF process [3,7].

Several methods are currently under development for reliable in-situ monitoring based on the sensors for defect detection and location [8,9,10,11,12,13,14,15,16]. The sensors applied for in-situ monitoring during the FFF process contain acoustic emission (AE) [8], accelerometer sensors [10], infrared temperature sensors [11], fiber Bragg grating (FBG) sensors [15], visual camera [16], and more. According to previous studies, the objectives of in-situ monitoring and diagnosis of the FFF process can be divided into two groups: one for the states of the FFF machine [8,9,10,11,12,13], and the other for the quality of the building parts [14,15,16,17,18,19].

The studies on the states of the FFF machine mainly focus on the plugged nozzle, broken or depleted filament, and driving belt fracture. Wu et al. [8,9] developed an AE monitoring method based on the support vector machine (SVM) and hidden semi-Markov model (HSMM) for identification of normal state, run-out-of-material state, and nozzle plugged state of the FFF process. Kim et al. [10] presented a system containing three accelerometers and an AE sensor to diagnose the loosened bolt in the nozzle head; the SVM-based models for the odd- and even-numbered layers of one FFF specimen are developed and the diagnosis accuracy is better than 87.5%. Liu et al. [11] introduced a data-driven predictive modeling approach to predict surface roughness of manufactured parts. Features in the time and frequency extracted by multiple sensor including thermocouples, infrared temperature sensors and accelerometers, was analyzed by six different machine learning algorithms. Yang et al. [12] used an AE sensor for filament breakage monitoring and found that the instantaneous skewness could be used as a preliminary indicator for filament breakage. Filament jam is also a common failure in FFF machines [20]. The reasons they jam are various, such as damage, stress, dust, and small debris in the filament, a wrong filament diameter, a breaking of the filament, or simply that the filament coil is over [21]. Soriano Heras et al. [20] presented a system to detect whether the filament was moving forward properly during the FFF process. The filament jam could be defected timely and the extrusion speed would be adjusted once the adjusted speed unmatching with the real speed. Fiedler et al. [22] and Volpato et al. [23] optimized the grip force on the 3D printer filament and developed novel feeding mechanisms, which effectively avoided the filament jam occurrence.

Previous works have proved that the quality of the products has been affected by various factors, such as residual stress [14,15,24,25,26,27,28,29,30], parameters settings [16,17,18,19], or vibration induced by external excitations [17]. The residual stress, which leads to delamination, warpage, and distortion during the FFF process, has been studied [14,15,24,25,26,27,28,29,30]. Fang et al. [14] embedded FBG sensors inside a 3D printing structure to monitor the strain variation and the measured results using FBG highly accorded with the theoretical results. Kousiatza et al. [15] used FBG sensors and thermocouples for real time monitoring of the generated residual strains and the results showed that FBG sensors were a reliable choice for the printed part’s quality during the FFF process. Kantaros et al. [24] used FBG sensors to investigate the magnitude of the post-fabrication residual strains considering two important parameters (layer thickness and deposition orientation). Wang et al. [25] introduced a mathematical mode to study warpage in the FFF process and found that extruded material was cooled from glass transition temperature to chamber temperature resulting in the development of inner stresses. Hammond et al. [26] presented the use of a vision system as a non-destructive in-situ monitor technique and the method showed that the defects caused by residual stress, such as warpage and delamination, can be detected effectively. Casavola et al. [27] employed the hole-drilling method to measure the residual stress in FFF parts and the results confirmed that good residual stress management could reduce the warpage and delamination in the finished part. Schimpf et al. [28] improved filament fusion and adhesion with the help of hydroxyl groups and their hydrogen bonding, which reduced the probability of warpage. Panda et al. [29] introduced an evolutionary system identification approach to explicitly quantify the warp deformation based on the line width compensation, extrusion velocity, filling velocity, and layer thickness, and found that the layer thickness and extrusion velocity influenced the warp deformation. Jang et al. [30] analyzed the accuracy of 3D-printed casts with Tukey honestly significant difference (Tukey HSD) test and they found that the residual internal stress may be generated when parameters were changed, which lead to possible deformation of the prosthesis.

Inappropriate parameter settings could bring building defects during the FFF process, such as large pores [17], rough surface [18,19], and so on. Greeff et al. [16] proposed an optical camera to monitor the filament fate rate and filament shredding could occur once the force required becomes too high. Du Plessis et al. [17] used X-ray computer tomography to monitor the quality of filament and found that the presence of large inclusions could affect the nozzle blockage or large pores in build parts. Rao et al. [18] used a heterogeneous sensor array including thermocouples, accelerometers, an infrared temperature sensor, and a real-time miniature video borescope to detect the defects of build parts, such as surface roughness. Sun et al. [19] proposed infrared ray (IR) temperature sensor and thermocouple to monitor the dimensional accuracy and surface roughness based on the functional quantitative and qualitative model. The effectiveness of the proposed method was demonstrated by simulation studies and experimental data. Ertay et al. [31] presented an integrated methodology to control the tangential path velocity, material deposition rate, and extruder temperature, the experiment results showed that the material was deposited more uniformly at sharp curvatures and the dimensional accuracy of printed parts was improved. Anderegg et al. [32] introduced a novel FFF nozzle that achieved in-situ monitoring. The flow temperature and pressure during extrusion, as well as a number of important insights into the printing process, were provided by the novel nozzle, which are vital for improving FFF printed parts. Go et al. [33] studied the FFF build rate, which was influenced by three main modules of FFF systems (the material extrusion, material heating, and positioning modules), suggesting that significant enhancements in FFF build rate with targeted quality specifications were possible via mutual improvements to the extrusion and heating mechanism along with high-speed motion systems.

To handle the large amount of data from sensors, data-driven methods are used owing to its flexibility in modeling, fast processing of prediction, and consideration of defect formation mechanisms [34]. The data driven methods used in-situ monitoring and diagnosing during the FFF process contains K-means [11], SVM [8,10], HSMM [9], compressive sensing [13], unsupervised density-based clustering method [11], and so on. In this paper, two data driven methods—the least squares support vector machine (LS-SVM) and the back-propagation neural network (BPNN) model—are introduced for in-situ monitoring and diagnosing during the FFF process.

The monitoring systems mentioned previously focused on the use of sensors. However, there are issues when carrying out the monitoring with these systems. Firstly, the multiple sensors used in diagnosing single defect for FFF process are costly. Secondly, very few sensors can precisely monitor and recognize both machine states and product quality during the FFF process. Further, the correlation between the defects and the variation in the information corresponding to various defect formations is hitherto unclear.

To solve the above issues, a simple and effective approach based on vibration sensors is introduced for in-situ monitoring and diagnosing the machine state and product quality during the FFF process. For recognizing the states of FFF machine, there are three features: the root mean square (RMS), crest factor (CF), and Kurtosis index (KI). These features are extracted from the acceleration data of the build platform. The normal and filament jam states are identified by the LS-SVM and SVM models, respectively. For predicting the product quality during the FFF process, the acceleration data of four channels from both the extruder and build platforms are collected, and RMS, mean, and standard deviation (STD) are applied. The normal, warpage, and material stack are recognized with the help of the BPNN model. The proposed approach can potentially serve as a diagnostic and prognostic tool during the FFF process.

## 2. Methodology and Experimental Setup

**Figure 1:**
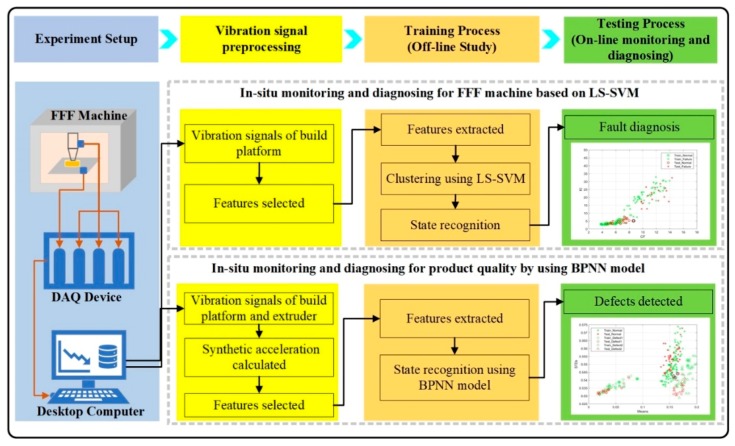
The flowchart of in-situ monitoring and diagnosing for fused filament fabrication (FFF) process. BPNN: back-propagation neural network; LS-SVM: least squares support vector machine; DAQ: data acquisition.

The methodology and the experimental setup proposed in this study are detailed in this section and the corresponding flowchart is shown in Figure 1. The basic steps for in-situ monitoring and diagnosing for the FFF process are as follows: (1) experiment setup, (2) vibration signal preprocessing, (3) training process, and (4) testing process. In step 1, the experiment system has been established by the FFF machine, vibration sensors, data acquisition (DAQ) device, and desktop computer. In step 2, the signals are collected and the features are selected. In steps 3 and 4, both the machine state and product quality are monitored and diagnosed with data driven models.

### 2.1. Experimental Setup

**Figure 2:**
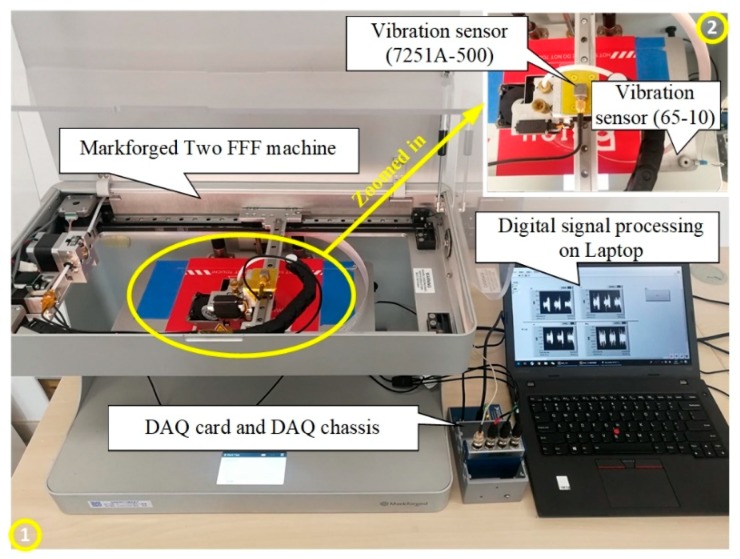
Vibration sensors based in-situ monitoring and diagnosing system during the FFF process.

The established system consists of the FFF machine, vibration sensors, DAQ system, and a desktop computer, as shown in Figure 2. The FFF machine used in this study is Markforged Two, a high-performance fiber composite FFF machine made by Markforged, (Watertown, MA, USA). The vibration signals are collected by two piezoelectric vibration sensors made by Endevco (Sunnyvale, CA, USA). One vibration sensor (7251A-500, single channel) with 500 mV/g output has been mounted on the build platform and the other (65-10, three channels) with 10 mV/g output is attached on the extruder. The signals are processed by a DAQ card, NI 9234 and a compact DAQ chassis, NI cDAQ-9174 made by National Instruments (NI), (Austin, TX, USA). The real time signals can be processed by a digital signal processing module on the laptop. The laptop used is Thinkpad T460p made by Lenovo and the signal processing software here is LabVIEW 2014. All data training and testing process is performed in a MATLAB environment. The specification of the FFF process in this paper is listed in Table 1. The Onyx in Table 1 is nylon mixed with chopped carbon fiber, which offers a high-strength thermoplastic with excellent heat resistance, surface finish, and chemical resistance, the heat deflection temperature of Onyx is 145 °C. The filling feed rate is 40 mm/s, the outer contour feed speed is 30 mm/s, and the inner contour rate is 18 mm/s. All of the three feed rates are calculated by dividing distance by time.

### 2.2. Vibration Signal Preprocessing

To obtain satisfactory results from the analyzing vibration signal, three factors need to be considered in practice.

Firstly, the quality of the signal is very important. Good signal acquisition accounts for more than 70% of success [35]. To improve the quality of the signals and ensure the repeatability of the results, mature and stable products are selected for the equipment listed in this paper, such as the FFF machine, vibration sensors, and DAQ system. The dimensions of the specimens used in this paper are according to ISO 178 [36].

Secondly, it is important to determine the right sampling rate for digitizing the vibration signal. The sample rate should be large enough to cover maximum useful information. According to the Nyquist theorem, the sampling frequency should be at least twice the maximum frequency present in the vibration signal. Nevertheless, excessive sampling rate may increase the cost of computation. Hence a sampling rate of 2.5 KHz has been used in the experiments to balance the information integrity with the computational efficiency.

Thirdly, to distinguish different operating states during FFF printing, the extracted features should be highly sensitive to operating conditions instead of being insensitive to noises. In this work, five commonly used features in time-domain signal, including mean, STD, RMS, CF, and KI have been introduced. For a vibration signal, x(n), let *N*, u, and σ denote the value of length, mean, and STD, respectively. Then RMS, CF, and KI can be expressed as follows.

- RMS is proportional to the energy contents of the signal in time domain, whose changes might signify the change of the 3D printer operating states, or it can be related to product defects.
  (1) $$RMS=\sqrt{\frac{1}{N}\sum_{i=1}^{N}{\left(x\left(i\right)\right)}^{2}}$$
- CF is the ratio of peak-to-valley value to the RMS value of the vibration signal and elucidates any outcome present in the vibration signal [37].
  (2) $$CF=\;\frac{max(x\left(n\right))-min(x\left(n\right))}{RMS}$$
- KI is the fourth standardized moment, which measures the degree of “peakiness” of a distribution compared to a normal distribution. Here, KI can be used as an indicator to measure the degree of vibration signal amplitude [37,38].
  (3) $$KI=\frac{\frac{1}{N}\sum_{i=1}^{N}{\left(x\left(i\right)-u\right)}^{4}}{\sigma^{4}}$$
  where, $u=\frac{1}{N}\sum_{i=1}^{N}x\left(i\right)$, $\sigma =\sqrt{\frac{1}{N}\sum_{i=1}^{N}{\left(x\left(i\right)-u\right)}^{2}}$.

### 2.3. In-Situ Monitoring and Diagnosing for the FFF Machine Based on LS-SVM

**Figure 3:**
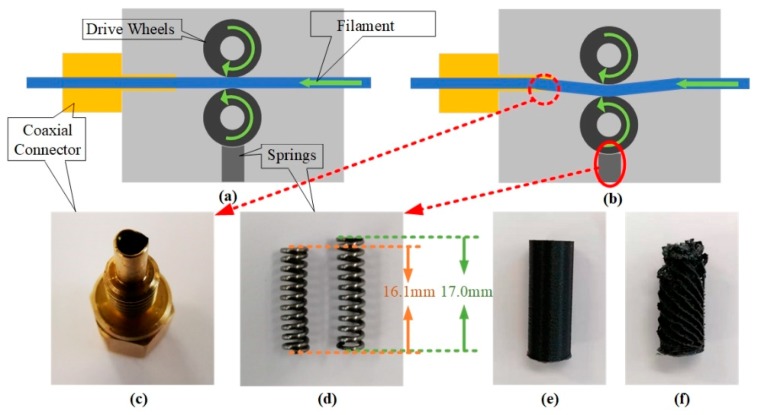
The failure mechanism of the filament jam: (**a**) the normal state; (**b**) the filament jam state caused by the increasing friction force between the filament and the coaxial connector; (**c**) the worn coaxial connector; (**d**) the fatigued spring (16.1 mm) and the normal spring (17.0 mm); (**e**) the normal specimen; (**f**) the specimen built in the filament jam state.

In this experiment, we studied filament jam, one of typical failure states of the FFF machine. In its normal state, the spring supports the drive wheels, which aligns the filament and the coaxial connector in a line (see Figure 3a). Once the spring fatigues the drive wheels sink, which leads to the friction force between filament and the coaxial connector increasing. Then the failure mode, i.e., the filament jam, is generated (see Figure 3b). The worn coaxial connector is shown in Figure 3c and the fatigued spring is shown in Figure 3d. From Figure 3d, we can learn that the length of normal spring is 17.0 mm, while the fatigued spring in this experiment is 16.1 mm. The specimen (⌀12 mm × 30 mm) have been designed with CATIA V5 and then transformed into STL files. The specimen built in the normal state is shown in Figure 3e, while the specimen in Figure 3f has been built in filament jam state.

The vibration sensor attached on the build platform has been used to diagnose the filament jam mode, considering that the filament jam easily causes the motor to be out of step, and the abnormal vibration that the FFF machine generates, including the build platform. In addition, the filament jam mode makes the mount of the filament for the extruder decrease, which reduces the force between the extruder and the building part. Then the amplitude of the build platform decreases accordingly.

In this paper, the normal and filament jam states are identified and recognized by the LS-SVM algorithm [39]. LS-SVM, like SVM [40], is based on the margin maximization principle, which performs structural risk minimization. LS-SVMs are closely related to regularization networks and Gaussian processes, but additionally emphasize and exploit primal-dual interpretations [41,42]. SVM has been adopted to identify the FFF machine conditions and it achieves good results [8,10]. However, the computational cost of SVM is high due to the constrained optimization programming [42], especially when the data is very large. In comparison to SVM, LS-SVM requires only the solution to a convex linear problem (a quadratic problem in SVM), which simplifies the solution of Lagrange multipliers and reduces the computational cost [43]. In view of this, the LS-SVM has been introduced for in-situ monitoring and diagnosing for the FFF machine.

The process of in-situ monitoring and diagnosing for FFF machine is given below (Figure 1). Firstly, the acceleration signals of the built platform are collected from the vibration sensor. Then, the off-line study is implemented. To improve the accuracy of recognition, the efficient features that are highly sensitive to the operating states of the FFF machine are selected. Subsequent to the features selection, LS-SVM with the radial basis function (RBF) kernel is used to classify the normal and abnormal states, and the classification results are compared with SVM. Lastly, online testing data are used to demonstrate the performance of the LS-SVM model.

### 2.4. In-Situ Monitoring and Diagnosing for Product Quality Using the BPNN Model

In the FFF process, the product quality depends on two fundamental parameters: the strength of the filament extruded by the heated nozzle and the strength of each of the extruded filament bonds in horizontal and vertical directions [44]. Generally, the strengths of extruded filaments and their mutual bonding are affected by the state of the FFF machine. However, quality defects may appear even when the FFF machine runs normally. The defects of the production are still generated because of the inaccurate adjustments of printer settings, chamber temperature abnormality, or the vibration induced by the external excitations. Warpage and abnormal leakage are two typical production defects.

**Figure 4:**
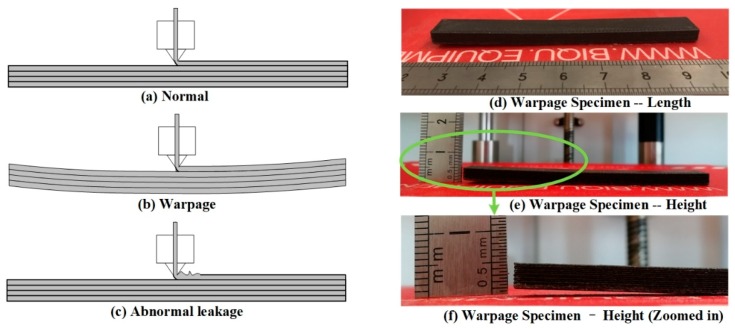
Normal, warpage, and material stack caused by abnormal leakage: (**a**) the normal; (**b**) warpage; (**c**) abnormal leakage; (**d**) the length of warpage specimen; (**e**) the height of warpage; (**f**) the zoomed-in warpage.

Warpage reduces the vibration strength of the build platform and the influences of the frictional forces between the extruder and specimen. The defect of the warpage has been developed by the residual strains or due to the stresses caused by the non-uniform thermal gradients [45], Abnormal leakage leads to material stack on the specimen, which causes abnormal vibration for the extruder and build platform. Abnormal leakage can be caused by the prolonged heating of the extruder or the degradation of the composites due to moisture. The specimen size is 80 mm × 10 mm × 4 mm and its geometric models have been designed according to the ISO 178. The reason to choose the specimen is that the warpage is easily discovered compared to other parts we used. As for Markforged Two (the FFF machine used in this experiment), the temperature and feed rate cannot be modified by users. After repetitious experiments, we find that when the fill rate is 100% and layer thickness is 0.2 mm, the warpage could occur. To identify the warpage and abnormal leakage, both the vibration sensors mounted on the build platform and extruder are used (see Figure 1). In this experiment, fault injection method is introduced for the abnormal leakage (see Figure 4c), and the length of the material stack caused by abnormal leakage is about 5 mm. Warpage is shown in Figure 4b and the specimen with warpage is shown in Figure 4d,f. The warpage in this experiment is 1 mm/80 mm (Figure 4d,f).

BPNN [46] is a network that utilizes the errors, owing to the discrepancy between the predicted outputs, desired outputs, and gradient descent, which realize the modifications for the connection weight. The modification for the connection weight of the network is targeted for minimizing errors. The fact that a single hidden layer BP network can approximate any measurable function arbitrarily has been proven in [47], wherein a larger number of hidden neurons brings a higher capability in approximating the complex relationships. However, having a larger number of hidden neurons is not necessarily desirable since two drawbacks will appear as the number grows [48]. The first one is overfitting, where once the prediction is outside the training domain, the results differ more significantly. The second is an increase in the computation burden. More unknown parameters need to be optimized if the number of hidden neurons grow, which may significantly increase the computational cost.

The process of in-situ monitoring and diagnosing for product quality during the FFF process is shown in Figure 1. Firstly, the vibration signals are collected and features extracted. The acceleration signals of extruder and built platform are collected. Before the features are extracted, the dimensions of signals are reduced and the synthetic acceleration (SA) is calculated. Then, during the training process, the efficient features that are highly sensitive to the defects are selected for building the product during 3D printing. The BPNN model are trained to identify the defects of building parts with the selected features. The k-fold cross validation (k-CV) method has been proposed to help determine the optimized number of hidden neurons and avoid overfitting to certain extent. Lastly, the trained BPNN model is used to predict the defects of building parts during the FFF process.

## 3. Results and Discussion

Experimental results during the FFF process have been analyzed in this section. In Section 3.1, the failure state and filament jam are diagnosed based on the LS-SVM model. In Section 3.2, the detection of the two common defects, warpage and abnormal leakage, using the BPNN model are detailed.

### 3.1. The Study of Fault Diagnosis for FFF Machine

#### 3.1.1. Signal Processing and Feature Extracted

**Figure 5:**
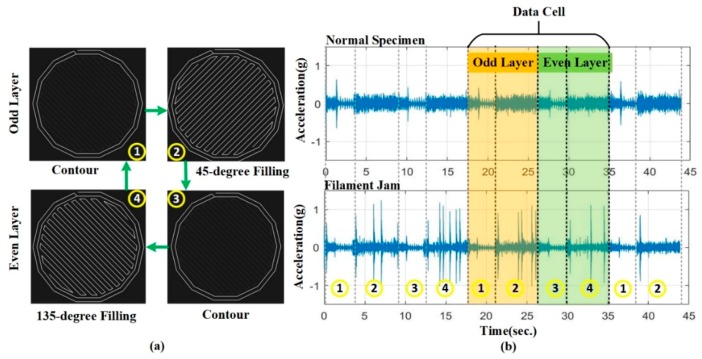
The basic working conditions and the length of the data cell: (**a**) the basic conditions; (**b**) the data cell and the signals for normal and filament jam states.

The acceleration signals of the build platform have been collected using mounted vibration sensors (Figure 2). Further, the specimens built in this experiment have been shown in Figure 3. Before extracting features, the length of data cells has been initially determined. The data cell should contain the basic working conditions to maximally cover the signal information. The basic working conditions have been illustrated in Figure 5. The odd-layer printing process contains two basic working conditions: contour and 45-degree filling (see Figure 5a) The contour and 135-degree filling are contained in the even-layer printing process (see Figure 5a). Therefore, there are three basic working conditions in the FFF process, viz., contour, 45-degree filling, and 135-degree filling. To cover these conditions, a data cell has been set as a continuum between the odd-layer and even-layer data regions (see Figure 5b). The vibration signals for both the normal and the filament jam states have been recorded, which is shown in Figure 5b, where the upper signal represents the normal state whereas the lower one represents the filament jam state.

The signal for filament jam has more abrupt changes and smaller amplitude in comparison with the normal state signal of the build platform. Both the CF and KI can be used as the indicators for measuring the degree of abrupt changes, whereas RMS can reflect the energy contents of the signal. In this perspective, the RMS, CF, and KI are selected as the features.

**Figure 6:**
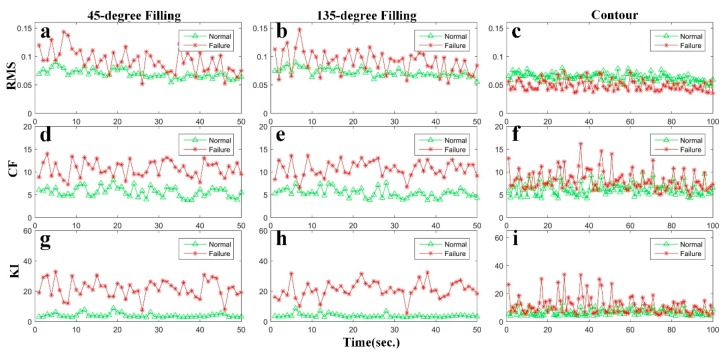
The values of (RMS), crest factor (CF), and Kurtosis index (KI) for training data. The red lines represent the filament jam state, the green lines represent the normal state: (**a**)–(**c**): the RMS values; (**d**)–(**f**) the CF values; (**g**)–(**i**) the KI values.

The two categories of specimens have been built by the FFF machine in normal and filament jam states. For each specimen, there are almost 360 million data collected in 25 min. The total layers for each of the specimen are 150. The first 100 layers are set as the training group, while the data from layer 101 to 140 are set as the testing group. The number of data cells in the training group are 50 and the number in testing group is 20. Each cell has one 45-degree filling, one 135-degree filling and two contours. The values of RMS, CF, and KI for the training data are shown as Figure 6. In each panel of Figure 6, the red lines represent the faulty state, whereas the green lines represent the normal state. The panels in the first column of Figure 6 is for 45-degree filling, the middle column for 135-degree filling, and the last column for contour.

To determine whether there are differences in the values of RMS, CF, and KI for normal and filament jam states, a one-way analysis of variance (ANOVA) followed with a Tukey HSD test at the 0.05 probability level is conducted to identify significant differences among the filling and contour conditions.

In Table 2, the mean and STD values of RMS, CF, and KI for normal and filament jam states are calculated. For 45-degree filling and 135-degree filling conditions, the mean values of RMS, CF, and KI in normal state are smaller than that in filament jam state, so do the STD values. For contour conditions, except that the mean value of RMS in normal state is bigger than that in filament jam state, both mean and STD values in normal state are smaller than that in filament jam state. These results are in accord with the abrupt changes of the signals for filament jam state. The p-values (p≤0.5, which is considered significant) of all pairs for normal and filament states in Table 2 are nearly zero, which means that the mean values of RMS, CF, and KI for normal and filament jam states are significantly different across normal and filament states.

#### 3.1.2. Filament Jam Diagnosis Based on LS-SVM

Following the extraction of features, both SVM and LS-SVM are applied for the state identification. The effect of the penalty parameter C in SVM and the regularization parameter γ in LS-SVM is similar, thus determining the trade-off between the fitting error minimization and smoothness. Considering the ability of nonlinear classification, the RBF has been chosen as the kernel function [48,49]:

(4) $$K\left(x_{1},x_{2}\right)=exp(-\frac{|\left|x_{1}-x_{2}\right|{|}^{2}}{2\sigma^{2}})$$

where, $\sigma^{2}$ is the bandwidth that controls the scaling ratio.

Here, a grid search is employed to determine the value of the parameters C, γ and σ. The value of C and γ has been selected as five and the value of σ as two. To avoid the over-fitting problem, K-fold cross-validation method has been introduced to test the SVM and LS-SVM models, and the value of k is ten.

The results of the state identification for the training group using SVM and LS-SVM, respectively, have been shown in Table 3. To the 45-degree filling, the results from the CF and KI are better than that of the RMS. Nevertheless, the accuracy in using SVM and LS-SVM are nearly the same, except the RMS whose results using LS-SVM has been more accurate (82% > 80%). To the 135-degree filling, the CF and KI also have better performance than the RMS, and the accuracy of the RMS (81%) and KI (99%) using LS-SVM is higher than SVM (80% and 98%). To contour, the RMS performs better than the CF and KI, and the result of the CF (73%) using LS-SVM is more accurate. From the above analysis, we find that LS-SVM performs better than SVM and the results of the 45-degree and 135-degree fillings are more accurate than that of the contour.

**Figure 7:**
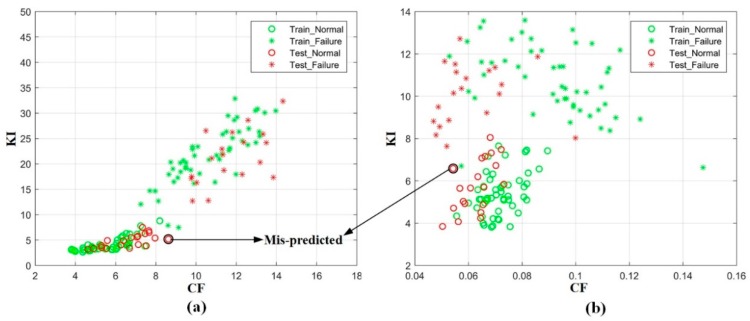
The results predicted using the multi-features based on the LS-SVM: (**a**) the 45-degree filling; (**b**) the 135-degree filling.

Considering the improved performance, the LS-SVM model has been used for in-situ monitoring and diagnosing for the FFF machine. In general, this single feature reflects less information in comparison with the multiple features. To improve the accuracy of the fault diagnosis, the RMS, CF, and KI are together taken as inputs for the LS-SVM model. The results of state identification for training group and testing group are shown in Table 4. The calculations of the 45-degree filling, 135-degree filling, and contour for the training group are 100%, 99%, and 81%, respectively. Comparing the results in Table 3 and Table 4, the LS-SVM model with multiple inputs is more accurate than the model with a single input. Using the training model, the results of 45-degree filling, 135-degree filling, and the contour, for testing the group are 97.5%, 97.5%, and 91.25%, respectively. The results of the prediction are shown in Figure 7. The green markers represent the cells from the training set and the red markers represent cells from the testing set. The circles represent the cells from normal state and the stars represent the cells from the filament jam state. The results of the 45-degree filling are shown in Figure 7a, whereas the results of the 135-degree filling are shown in Figure 7b, beside the markers circled by black circles, which represent mis-predicted points.

### 3.2. The Study of Defects Detected for Specimens

#### 3.2.1. Signal Processing and Feature Extracted

**Figure 8:**
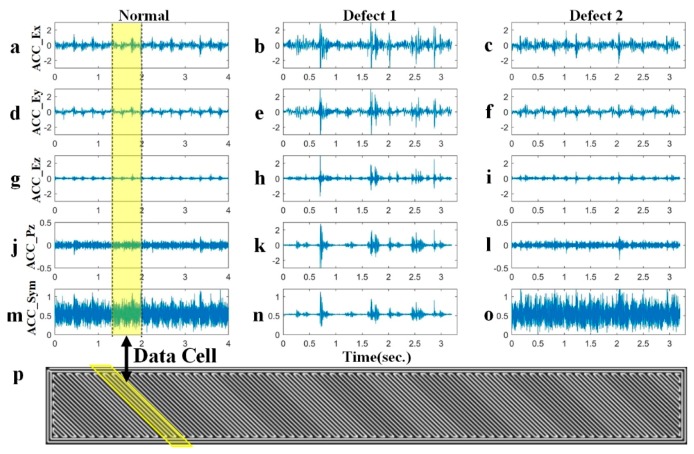
The acceleration signals: (**a**)–(**c**) x direction of the extruder; (**d**)–(**f**) y direction of the extruder; (**g**)–(**i**) z direction of the extruder; (**j**)–(**l**) z direction of the build platform; (**m**)–(**o**) synthetic acceleration; (**p**) the basic working condition and the yellow zone stands for the data cell.

To diagnose the defects of the building products during the FFF process, the acceleration signals are collected from the two installed vibration sensors as shown in Figure 2. There are four signal channels: one is from the sensor mounted on the build platform and the other three are from the sensors attached to the extruder. The examples of the four-channel signals are shown in Figure 8. The three columns in Figure 8 denote the signal of normal, warpage (defect 1), and material stack specimens caused by the abnormal leakage (defect 2), respectively. The first three rows (Figure 8a–i) display the vibration signals of the extruder in the x, y, and z directions, respectively, and the fourth row (Figure 8j–l) is the signals in the z-direction for the build platform.

The synthetic acceleration (SA) has been employed to reduce the computational complexity. When compared with the uniaxial acceleration (UA), the SA can reveal more information. The SA is defined as:

(5) $$a_{Ei}=\sqrt{{\left(a_{Ei}^{x}\right)}^{2}+{\left(a_{Ei}^{y}\right)}^{2}+{\left(a_{Ei}^{z}\right)}^{2}},$$

(6) $$a_{i}=\sqrt{{\left(normalize\left(a_{Ei}\right)\right)}^{2}+{\left(normalize\left(a_{Bi}\right)\right)}^{2}},\;i=1,\;2,\;3,\ldots ,n$$

where $a_{Ei}$ is the SA for the extruder, $a_{Ei}^{x}$, $a_{Ei}^{y}$, and $a_{Ei}^{z}$ are the UA of x, y and z directions for the extruder respectively, i∈{1, 2, 3,…,n} is the data number, $a_{Bi}$ is the UA of z-direction for build platform, and $a_{i}$ is the SA for $a_{Ei}^{x}$, $a_{Ei}^{y}$, $a_{Ei}^{z}$, and $a_{Bi}$.

The comparison between the SA signals of the normal and abnormal specimens are shown in Figure 8m–o. The SA signal for material stack has smallest amplitude in the three SA signals, whereas the SA signal for warpage has the biggest amplitude. Therefore, the features closely relate to the mean, STD, and RMS can be used as the candidates. In this experiment, the mean, STD, and RMS are selected as the features.

Considering the fill style for building the specimens as shown in Figure 8, the length of the data cell (marked by the yellow zone in Figure 8p) has been determined as the two round-trips of the extruder, whereas there are 1120 data elements in a cell. The step length used in this experiment is set as one round-trip with 560 data elements in a step.

#### 3.2.2. Multi-State Identification Based on BPNN

In this section, the BPNN model for a single hidden layer is selected to monitor and diagnose the quality defects. Three data groups are used in the training process. The first group is for the normal specimens where 90 data cells are obtained from 110,000 data elements. The second group is for the warpage specimens where 50 data cells are obtained from 60,000 data elements. The third group is for the material stack specimens caused by abnormal leakage, where 60 data cells are obtained from 70,000 data elements. To ensure the effectiveness and credibility of the defect identification, all the data used here are collected from Layer 10 (a total of 20 layers).

The k-CV method has been introduced to help determine the number of hidden neurons for the BPNN and to avoid overfitting to a certain extent. The inputs of BPNN model contain the mean, STD, and RMS. The outputs of BPNN model contains the normal, warpage, and material stack, and the output format is shown in Table 5. Here, the value of *k* in k-CV is set as 10 and the number of hidden neurons has been determined as four after cross-validation.

Three data groups are used in the testing process. The first group is for the normal specimens where 45 data cells are obtained from 55,000 data elements. The second group is for the warpage specimens where 25 data cells are obtained from 30,000 data elements. The third group is for the material stack specimens caused by abnormal leakage where 30 data cells are obtained from 35,000 data elements.

The results from the comparison for the SA and UAs are shown in Table 6. For the first data group, the accuracy rate for the SA is 95.56%, which is equal to the results for the z-axis acceleration for extruder, but better than the results (84.4%, 93.3%, and 93.3%) for the other three UAs. For the second data group, the accuracy rate for the SA is 96%, which is better than the results (84%, 68%, 72%, and 80%) for the four UAs. For the third data group, the accuracy rate for SA is 100%, which is equal to the results for extruder z-axis acceleration, but better than the results (96.67%, 93.3%, and 93.3%) for the other three UAs. For the total of three data groups, the accuracy rate for the SA is 97%, which is better than the results (88%, 87%, 91%, and 90%) for the four UAs. Considering the results in Table 6, the BPNN model with SA performs better than that with UAs.

**Figure 9:**
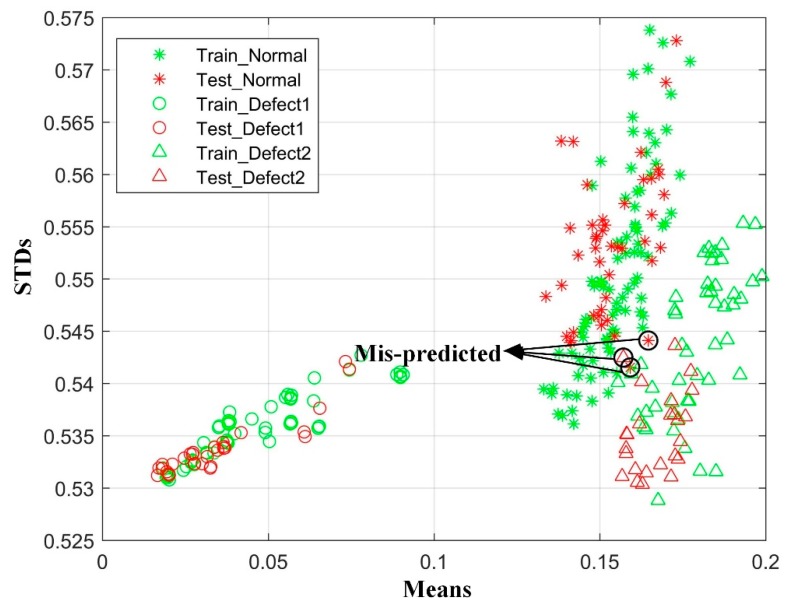
The predicted results for SA, where the green markers represent cells from the training set and the red markers represent the cells from the testing set. The stars, circles, and triangles represent the cells from the normal state, warpage, and material stack. The markers circled by black circles are mis-predicted.

The predicted results using the BPNN model with SA is shown in Figure 9: defects 1 represents the material stack caused by abnormal leakage and defects 2 for the warpage. The markers circled by black circles are mis-predicted points. Figure 9 shows that the warpage markers are closer to normal markers, which implies that the degree of vibration for normal and warpage are nearly the same.

## 4. Conclusions

In this paper, an approach based on the vibration sensors and data-driven methods has been introduced for in-situ monitoring and diagnosing during the FFF process. Both machine states and production quality are monitored for the sake of high product quality.

The normal and filament jam states are effectively recognized by both SVM and LS-SVM, while LS-SVM achieves better performance. The LS-SVM model with multiple inputs is more accurate than the model with a single input. Using the training model, the testing results of 45-degree filling, 135-degree filling, and the contour, for testing the group are 97.5%, 97.5%, and 91.25%.

The normal, warpage and material stack are predicted with the help of the BPNN model. Both the UA and SA are used for predicting the defects during the FFF process where SA gets a better accuracy rate than UAs. The predicted results of normal, warpage and Material stack using the BPNN model with the SA are 95.56%, 96%, and 100%.

The study presents a useful method to in-situ monitor and diagnosis for the FFF process. However, limits exist in this study. Firstly, the vibration intensity is strongly affected by the feed rate. Owing to restrict of the close-loop process of the FDM machine used in this work, the feed rate cannot modify. Secondly, warpage and abnormal leakage is indeed found with vibration sensors, but the formation mechanisms for the defects are not researched. Therefore, further investigations will be centered on following areas. Primarily, the FFF machines with open parameters settings are given priority. The filament jam would be further studied with different parameter settings, such as feed rate, extruder temperature, and so on. Secondarily, the residual stress and extruder temperature would be in-situ monitored and studied to find out the formation mechanisms of warpage and abnormal leakage during the FFF process.

## Figures and Tables

**Table 1:** Specification of the FFF process.

Type	Value
Material	Onyx
Extruder temperature	265 °C
Nozzle diameter	0.4 mm
Layer thickness	0.2 mm
Filling Density	100%
Filling Pattern	Rectangular
Filling feed rate	40mm/s
Contours	2
Contour feed rate	30 mm/s (outer), 18 mm/s (inner)

**Table 2:** The mean and STD values for RMS, CF, and KI, and *p*-values of all pairs for normal and filament states.

Working Condition	State	Cell Numbers	RMS			CF			KI		
			Mean	STD	*p*-Value	Mean	STD	*p*-Value	Mean	STD	*p*-Value
45-degree filling	Normal	50	0.0695	0.0075	8.13 × 10^−10^	5.61	1.10	1.06 × 10^−10^	4.03	1.35	1.06 × 10^−10^
	Filament Jam	50	0.0926	0.0202		10.67	1.71		21.70	6.30	
135-degree Filling	Normal	50	0.0718	0.007	2.07 × 10^−9^	5.38	1.02	1.06 × 10^−10^	3.81	1.16	1.06 × 10^−10^
	Filament Jam	50	0.0914	0.0198		10.73	1.66		21.07	5.28	
contour	Normal	100	0.0636	0.0072	1.24 × 10^−10^	6.25	1.36	1.06 × 10^−10^	6.65	2.36	1.08 × 10^−10^
	Filament Jam	100	0.0487	0.0091		8.29	2.17		11.58	6.65	

**Table 3:** The results of state identification using SVM and LS-SVM for training group.

Features	45-Degree Filling		135-Degree Filling		Contour	
	SVM	LS-SVM	SVM	LS-SVM	SVM	LS-SVM
RMS	80%	82%	80%	81%	81%	81%
CF	97%	97%	98%	98%	72%	73%
KI	98%	98%	98%	99%	66%	66%

**Table 4:** The results of states identification using multiple features for both training group and testing group.

	Odd Fill	Even Fill	Contour
Training group	100%	99%	81%
Testing group	97.5%	97.5%	91.25%

**Table 5:** The outputs format of BPNN model.

Outputs	Value 1	Value 2	Value 3
Normal	1	0	0
Warpage	0	1	0
Material stack	0	0	1

**Table 6:** The predicted results for uniaxial acceleration (UA), and synthetic acceleration (SA) using the BPNN model.

Channel		Normal	Warpage	Material Stack	Total
**UA**	Extruder-x	84.4%	84%	96.67%	88%
	Extruder-y	93.33%	68%	93.33%	87%
	Extruder-z	95.56%	72%	100%	91%
	Platform-z	93.33%	80%	93.33%	90%
**SA**		95.56%	96%	100%	97%
